# Problematic generative AI use and mental health risks among Chinese university students: latent profiles, network structure, and health literacy as a modifiable resource

**DOI:** 10.3389/fpubh.2026.1910408

**Published:** 2026-08-20

**Authors:** Zeyu Zhang, Yinlan Zhang, Xiaomei Lu, Min Zhang

**Affiliations:** 1 Judicial Police College, Xinjiang University of Political Science and Law, Tumxuk, China; 2 Department of Medical Technology, Nanjing Second People's Hospital, Nanjing, China; 3 Medical Appraisal Center, Sihong Hospital, Suqian, China; 4 Teaching and Research Group, Suqian Chongwen Junior Middle School, Suqian, China

**Keywords:** generative artificial intelligence, health literacy, latent profile analysis, mental health, network analysis, problematic use, university students

## Abstract

**Objective:**

Generative artificial intelligence (GenAI) has become a routine academic tool for university students, but dysregulated use may co-occur with anxiety, sleep problems, psychological distress, and impaired academic functioning. This study examined GenAI-related digital behavior as a public health issue and assessed whether health literacy and self-regulated learning were associated with lower-risk profiles.

**Methods:**

A cross-sectional survey was conducted among Chinese university students from six universities in eastern, central, and western China. A total of 2,140 questionnaires were submitted; after four-step data-quality screening, 1,872 valid responses were retained. Measures assessed GenAI-use dysregulation, academic anxiety, sleep problems, psychological distress, health literacy, self-regulated learning, academic engagement, and demographic and use-related covariates. Latent profile analysis identified GenAI-use and mental-health risk profiles. Profile differences were examined using omnibus and *post hoc* tests, multinomial logistic regression examined external factors associated with profile membership, and regularized network analysis identified central and bridge nodes.

**Results:**

A four-profile solution showed the best balance of fit, entropy, class size, and interpretability (entropy = 0.891). The profiles were low-risk adaptive users (39.9%), anxiety-prone dependent users (25.7%), sleep-disrupted overusers (19.8%), and high-risk dysregulated users (14.6%). Confirmatory factor analysis supported the measurement structure of the adapted and selected scales (CFI = 0.958, TLI = 0.951, RMSEA = 0.036, SRMR = 0.041), and Harman's single-factor test did not indicate serious common method bias. Higher health literacy and self-regulated learning were associated with greater odds of low-risk rather than high-risk membership. Network analysis identified loss of control, academic worry, delayed bedtime, and academic avoidance as central or bridge nodes, whereas credibility evaluation and time management showed negative bridge expected influence.

**Conclusion:**

GenAI-related digital behavior among university students varied in ways that involved dysregulated use, academic anxiety, sleep problems, distress, and academic functioning. These findings may help universities identify students who report difficulty controlling AI use, academic worry, delayed bedtime, academic avoidance, or trouble setting limits on AI use. They also point to health literacy, self-regulation, and time management as candidate resources for future longitudinal and intervention studies.

## 1 Introduction

Generative artificial intelligence (GenAI), represented by large language models (LLMs), has quickly become part of university students' academic routines. Students use these systems to search for information, draft outlines, translate or polish text, summarize readings, check answers, and obtain feedback. These uses are not simply educational-technology practices; they also shape how young adults manage information, uncertainty, academic pressure, and everyday decisions in digital environments (1–4). Although LLMs can lower information barriers, concerns remain about hallucination, misplaced trust, overreliance, and weakened independent judgment (5–8). In health and medical contexts, LLMs are also being examined for information support and decision assistance, but their reliability and workflow integration remain under evaluation (9, 10).

For universities, GenAI therefore creates challenges beyond academic honesty and assessment design. Students may interact with AI tools when they are tired, anxious, uncertain, or isolated. Some may use GenAI to avoid difficult learning tasks, obtain reassurance, or continue working late into the night. The public health question is not whether students use GenAI frequently, but whether use becomes difficult to regulate, stress-maintaining, or linked with impaired sleep, mental health, and self-management.

Problematic GenAI use can be conceptualized as a specific form of dysregulated digital behavior. It may include loss of control over use, repeated unsuccessful efforts to reduce use, compulsive checking, excessive time spent interacting with AI tools, academic avoidance, reduced independent thinking, and emotional dependence on AI responses. This definition draws from the broader literature on problematic Internet use, smartphone overuse, and specific Internet-use disorders. At the same time, GenAI differs from earlier digital technologies in three important ways. First, it is conversational and can provide immediate reassurance when students feel uncertain. Second, it generates fluent and seemingly authoritative responses, which may increase misplaced confidence in unverified content. Third, it can complete or scaffold cognitive work, which may encourage avoidance of difficult thinking tasks when used without boundaries (11–17). Thus, the relevant mechanism may involve behavioral overuse, cognitive outsourcing, and anxiety-driven coping rather than mere frequency of tool access.

University students are a particularly relevant population for examining these risks. They are transitioning toward independent learning and self-management, while also facing academic pressure, career uncertainty, peer comparison, and increasing reliance on digital platforms. Mental health problems among university students have been widely documented, and anxiety, sleep disturbance, and psychological distress are closely linked to academic functioning and everyday health behavior (18–26). The rise of GenAI creates a new context for these concerns. On one hand, AI support may reduce immediate task burden and provide accessible academic assistance. Students who already experience academic anxiety may become more dependent on GenAI as a coping strategy. Students who use GenAI late at night or across long study sessions may also experience delayed sleep and daytime dysfunction. The question is therefore not whether GenAI is beneficial or harmful in general. A more useful question is which students show risk-prone patterns of use and which psychological or health-related indicators characterize those patterns.

Health literacy provides a relevant public health lens for this issue. Health literacy refers to the ability to access, understand, evaluate, and apply health information to maintain or improve health (27, 28). We focused on health literacy rather than AI literacy because the present study was framed around mental health, sleep health, and responsible health-related decision-making in digital environments, rather than general AI competence or technical AI knowledge. AI literacy is important for understanding and using AI systems, but health literacy is more directly related to evaluating health-relevant information, recognizing misleading or unsupported claims, and deciding when professional or institutional support is needed. In digital contexts, this ability increasingly includes recognizing information quality, evaluating credibility, distinguishing reliable guidance from misleading content, and translating information into appropriate behavior (29–35).

Most existing studies of digital behavior and student mental health remain variable-centered. They estimate average associations between use intensity, anxiety, sleep, and engagement. Such models are useful but may obscure meaningful heterogeneity. Students with similar levels of GenAI use may differ substantially. Some may be adaptive users with high self-regulation. Others may use GenAI mainly because they are anxious about academic performance. A further group may show sleep-related overuse, while a smaller group may present multiple concerns at the same time. Latent profile analysis (LPA) is well suited to identifying such person-centered patterns because it classifies individuals according to configurations of indicators rather than assuming a single average pathway (36–41). In addition, network analysis can identify central nodes and bridge nodes among symptoms, behaviors, and resources. It therefore moves beyond profile description toward candidate indicators that can be tested in later longitudinal or intervention studies (42–49).

Average-association models are also difficult to translate into practice. If problematic use is only reported as correlated with anxiety, it remains unclear whether universities should pay closer attention to anxiety, sleep, time management, AI literacy, or information-evaluation skills. Profiles can identify groups with different support needs, while network analysis can locate specific behaviors or symptoms that warrant closer follow-up.

**Figure 1:**
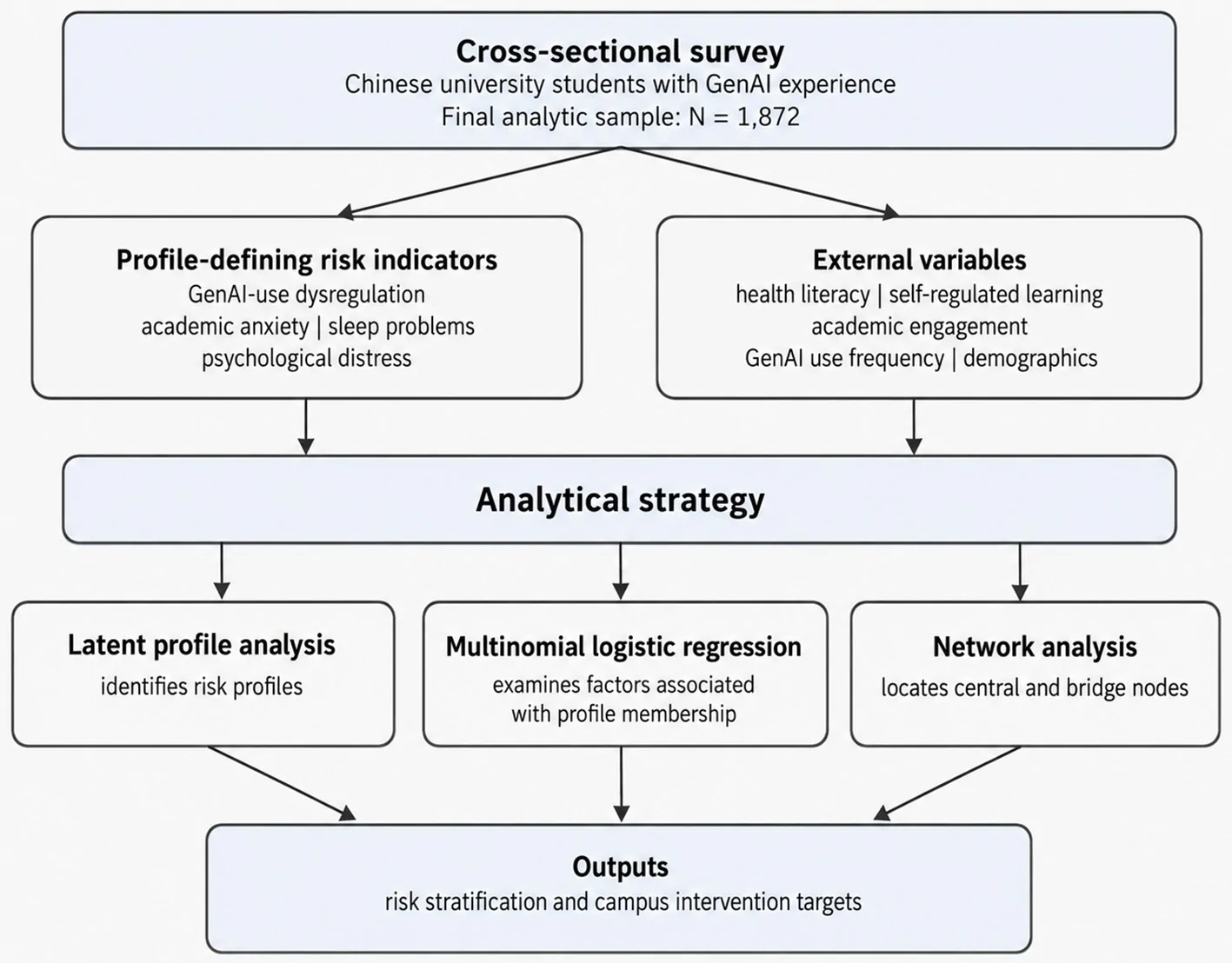
Study design and analytical workflow for identifying GenAI-use and mental-health risk profiles and network indicators.

The present study reframed problematic GenAI use as an emerging digital public health concern among university students and integrated LPA with network analysis. Because the latent profiles were defined by both GenAI-use dysregulation indicators and mental-health indicators, the resulting classes are interpreted as GenAI-use and mental-health risk profiles rather than profiles of GenAI use alone. We addressed three aims: to identify latent profiles of GenAI-use dysregulation and mental health risks; to compare health literacy, self-regulated learning, and academic engagement across profiles; and to identify external factors and network nodes associated with higher-risk membership. The study design and analytical workflow are shown in Figure 1.

## 2 Methods

### 2.1 Study design and participants

This cross-sectional survey examined GenAI-related behavioral dysregulation and mental health indicators among Chinese university students. Data were collected between April 20 and May 30, 2026, after ethics approval had been obtained. Participants were recruited from six universities located in eastern, central, and western China. Recruitment notices were distributed through course groups, student affairs offices, and online class communication channels. A convenience sampling strategy was used, with efforts to include students from different grades and disciplinary backgrounds.

Eligible participants were full-time undergraduate students aged 18 years or older who had used at least one GenAI tool for academic or personal learning purposes and voluntarily provided electronic informed consent. Students were excluded if they did not complete the core scales, failed the attention-check item, submitted implausibly short responses, or showed patterned responses indicating low engagement.

A total of 2,140 questionnaires were submitted. After data-quality screening, 268 responses were excluded and 1,872 valid responses were retained for analysis, yielding an effective response rate of 87.5%. The questionnaire recorded GenAI use frequency and main use purposes to distinguish general exposure from dysregulated or risk-prone use.

### 2.2 Procedure and data quality control

The questionnaire was administered online through a secure Chinese survey platform. The first page provided information about the study purpose, the voluntary nature of participation, anonymity, data confidentiality, and the right to withdraw before submission. Students who agreed to participate proceeded to the questionnaire. No personally identifying information was required for the analytic dataset, and responses were used only for research purposes.

The questionnaire included demographic information, GenAI-use characteristics, problematic GenAI use, academic anxiety, sleep problems, psychological distress, health literacy, self-regulated learning, and academic engagement. Items were arranged by construct and written in clear language appropriate for university students. The survey platform prevented submission unless all core scale items had been completed, thereby reducing item-level missingness.

Four data-quality procedures were applied before analysis. First, 96 incomplete questionnaires were removed. Second, one attention-check item was embedded in the questionnaire, and 54 responses that failed this item were excluded. Third, 68 questionnaires completed in less than 180 s were removed. This threshold was chosen because pilot testing indicated that careful completion of the core scales required at least 3 min, and response-time logs showed that such short submissions were unlikely to reflect careful reading. Fourth, 50 patterned responses, such as identical answers across long item blocks or visually repetitive response strings, were identified using the survey-platform export, SPSS screening, and R-based response-pattern checks. The final analytic sample comprised 1,872 valid responses.

### 2.3 Measures

All measures were self-report scales. Higher scores indicated higher levels of the measured construct unless otherwise specified. Scale domains, scoring approaches, example items, reliability coefficients, and source or adaptation information are summarized in Supplementary Table S1. The measures covered four domains: GenAI-use dysregulation, mental health indicators, modifiable resources, and academic functioning. The terms “risk indicators” and “resources” are used descriptively and do not imply causal effects in this cross-sectional study.

Before the main analysis, scale scores were examined for range, internal consistency, and distributional plausibility. Internal consistency coefficients ranged from 0.81 to 0.92, indicating acceptable to excellent reliability across constructs. Additional psychometric analyses, including confirmatory factor analysis, composite reliability, average variance extracted, and discriminant validity checks, were conducted for the adapted and selected scales and are reported in the Results and Supplementary Table S3.

#### 2.3.1 Problematic generative AI use

Problematic GenAI use was assessed with eight items adapted from problematic Internet use, generalized problematic Internet use, smartphone overuse, and technology-use frameworks and revised for the GenAI context (11–16). The item pool covered loss of control, failed reduction, compulsive checking, overreliance, academic avoidance, reduced independent thinking, emotional dependence, and excessive time use. Items were reviewed by two education researchers and two public health researchers for content relevance and clarity, and minor wording adjustments were made after pilot feedback from 32 undergraduate students. Items were rated on a five-point Likert scale, with higher scores indicating more dysregulated GenAI use. The total score was used in descriptive and external comparison analyses. To avoid repeated weighting in the latent profile model, the profile-defining GenAI indicators were represented by the core subdimensions of loss of control, compulsive use, and academic avoidance. The scale showed excellent internal consistency in this study (Cronbach's α = 0.91).

#### 2.3.2 Academic anxiety

Academic anxiety was measured using five items reflecting worry about academic performance, fear of falling behind classmates, anxiety when completing assignments or preparing for examinations, and nervousness during difficult academic tasks. The items were selected and adapted from student academic-anxiety and mental-health research to capture performance-related worry that is common in university settings (18–26). Items were rated on a five-point Likert scale. Higher scores indicated greater academic anxiety. Internal consistency was high (Cronbach's α = 0.90).

#### 2.3.3 Sleep problems

Sleep problems were assessed using a PSQI-type approach covering subjective sleep quality, sleep latency, sleep duration, sleep efficiency, sleep disturbance, daytime dysfunction, and delayed bedtime related to digital-device or GenAI use. This measurement focus is consistent with student sleep-health research and digital-use studies that link problematic technology use with delayed sleep and daytime impairment (21, 22). Higher scores indicated poorer sleep quality and more pronounced sleep-related problems. The scale showed acceptable internal consistency (Cronbach's α = 0.81).

#### 2.3.4 Psychological distress

Psychological distress was assessed using six items reflecting emotional exhaustion, mental overload, difficulty relaxing after academic tasks, irritability, reduced motivation, and perceived inability to cope with academic demands. These indicators were included to capture the broader distress component that may co-occur with academic anxiety and sleep problems in university populations (18–26). Items were rated on a five-point Likert scale, with higher scores indicating greater distress. Internal consistency was good (Cronbach's α = 0.88).

#### 2.3.5 Health literacy

Health literacy was measured using an eight-item short-form health literacy approach based on established definitions emphasizing access, understanding, evaluation, and application of health information (27–30). Because the study concerned GenAI use in a digital environment, additional attention was given to distinguishing reliable from misleading online information, making health-related decisions based on information, and managing personal health information. Health literacy was treated as an external modifiable resource rather than an AI-competence measure. Items were rated on a four-point response scale, with higher scores indicating stronger health literacy. Internal consistency was good (Cronbach's α = 0.89).

#### 2.3.6 Self-regulated learning

Self-regulated learning covered goal setting, time management, monitoring learning progress, strategy adjustment, and independent problem solving before seeking AI help. The construct was treated as an external modifiable resource rather than a profile-defining indicator. Items were rated on a five-point Likert scale. Higher scores indicated stronger self-regulated learning. Internal consistency was excellent (Cronbach's α = 0.92).

#### 2.3.7 Academic engagement

Academic engagement covered behavioral, cognitive, emotional, and interactive engagement. Academic engagement was treated as an external functioning variable used to interpret profile differences. Items were rated on a five-point Likert scale. Higher scores indicated greater academic engagement. Internal consistency was excellent (Cronbach's α = 0.90).

#### 2.3.8 Covariates

Covariates included gender, age, grade, discipline, residence, monthly living expenses, GenAI use frequency, and main GenAI use purpose. GenAI use frequency was categorized as rarely, 1–2 times per week, 3–5 times per week, or daily or almost daily. These covariates were included because demographic background and routine use patterns may be linked with both problematic use and mental health indicators.

### 2.4 Statistical analysis

Descriptive statistics were calculated for demographic variables and key study constructs. Categorical variables were summarized as frequencies and percentages, whereas continuous variables were summarized as means and standard deviations. Reliability was assessed using Cronbach's α. Pearson correlations were used to examine bivariate associations among core constructs. Group differences across latent profiles were examined using chi-square tests for categorical variables and one-way analysis of variance for continuous variables, followed by *post hoc* comparisons when appropriate.

Confirmatory factor analysis (CFA) was conducted to examine the measurement structure of the adapted and selected scales. Model fit was evaluated using χ^2^/df, comparative fit index (CFI), Tucker-Lewis index (TLI), root mean square error of approximation (RMSEA), and standardized root mean square residual (SRMR). Composite reliability (CR) and average variance extracted (AVE) were calculated to evaluate convergent validity, and discriminant validity was examined by comparing the square root of AVE with inter-construct correlations.

Because all variables were collected using self-report questionnaires in a single survey, common method variance was examined using Harman's single-factor test. All measurement items were entered into an unrotated exploratory factor analysis. Common method bias was considered less likely if the first factor accounted for less than 40% of the total variance.

The screened final dataset was used for all analyses. Missing data were minimal because the survey platform required completion of core scale items; no imputation was performed for core scale scores.

Latent profile analysis was conducted using six standardized profile-defining indicators: loss of control, compulsive GenAI use, academic avoidance, academic anxiety, sleep problems, and psychological distress. These indicators were chosen *a priori* because they represented the hypothesized co-occurrence of GenAI-use dysregulation and mental-health indicators. The total problematic GenAI use score was not entered together with its subdimensions in the LPA, which avoided repeated weighting of the same behavioral domain. Health literacy, self-regulated learning, and academic engagement were not used to form the latent profiles because they were conceptualized as external modifiable resources or functioning variables. This decision reduced circular interpretation and allowed the analysis to test whether these variables distinguished lower- and higher-risk memberships.

All profile-defining indicators were standardized before LPA so that measures with different response ranges contributed comparably to profile estimation. Because the LPA included both GenAI-use dysregulation and mental-health indicators, the resulting classes are interpreted as GenAI-use and mental-health risk profiles rather than profiles of problematic GenAI use alone.

After the optimal profile solution was selected, the average posterior classification probabilities were examined to assess classification precision. Mean differences in profile-defining indicators and external variables were then tested to support substantive interpretation of each profile. The selected labels were based on relative elevations and reductions across risk indicators, together with external differences in health literacy, self-regulated learning, and academic engagement.

Multinomial logistic regression was used to examine external factors associated with profile membership. The high-risk dysregulated profile was specified as the reference group because the main analytic interest was to determine which factors distinguished lower-risk or more specific risk groups from the most concerning profile. To avoid circular interpretation, variables used to define the latent profiles were not entered as predictors in this model. The model included gender, age, daily GenAI use, health literacy, self-regulated learning, and academic engagement. Results are reported as odds ratios (ORs) with 95% confidence intervals (CIs).

Network analysis was used to examine conditional associations among selected GenAI-use indicators, mental health symptoms, modifiable resources, and academic functioning. Nodes were selected *a priori* to represent the most conceptually central dimensions from each domain while avoiding an overly dense network with redundant item-level indicators. A regularized Gaussian graphical model was estimated using the extended Bayesian information criterion graphical least absolute shrinkage and selection operator (EBICglasso), with the EBIC hyperparameter γ set to 0.50. Analyses were conducted in R using the qgraph, bootnet, and networktools packages. Edges represent partial correlations after controlling for all other nodes in the network. Expected influence was used rather than strength centrality because the network included both positive and negative edges. Bridge expected influence was used to identify nodes that connect GenAI-use indicators with mental health indicators, resource variables, and academic functioning (43–46, 50).

Nodes were grouped into four conceptual communities: problematic GenAI use, mental health indicators, modifiable resources, and academic functioning. This four-community grouping followed the study framework and the measurement domains, rather than a *post hoc* search for the strongest visual separation. The grouping was used only for bridge-analysis interpretation; the network estimation itself was data-driven through regularized partial correlations. The network was visualized using qgraph with a Fruchterman-Reingold layout, and labels and colors were formatted only for readability without changing edge signs or relative edge-weight information.

Network accuracy and stability were evaluated using nonparametric bootstrapping and case-dropping bootstrapping. Edge-weight accuracy was evaluated through bootstrapped confidence intervals. Centrality stability was evaluated using correlation stability coefficients. Values greater than 0.25 were interpreted as acceptable and values greater than 0.50 as preferable for stable interpretation. Because the data were cross-sectional, network edges and bridge expected influence were interpreted as conditional associations and hypothesis-generating indicators rather than evidence of causal pathways or clinical intervention effects.

### 2.5 Ethical considerations

The study was conducted in accordance with the Declaration of Helsinki and was approved by the Institutional Ethics Committee of Xinjiang University of Political Science and Law, China (Approval No. XJPL2026010; approval date: April 15, 2026). Participants provided informed consent electronically before completing the questionnaire. Participation was voluntary, and students could exit the survey before submission. The dataset was anonymized before analysis, and no personal identifying information was used in the manuscript.

## 3 Results

### 3.1 Measurement quality, participant characteristics, and descriptive statistics

Additional psychometric analyses were conducted before the main substantive analyses. CFA supported the measurement structure of the adapted and selected scales, χ^2^/df = 3.42, CFI = 0.958, TLI = 0.951, RMSEA = 0.036, and SRMR = 0.041. Standardized factor loadings ranged from 0.63 to 0.88 across constructs. CR values ranged from 0.89 to 0.92, and AVE values ranged from 0.53 to 0.66, supporting convergent validity. The square roots of AVE were higher than the corresponding inter-construct correlations, supporting discriminant validity. Detailed results are presented in Supplementary Table S3.

Harman's single-factor test indicated that the first unrotated factor accounted for 28.6% of the total variance, below the commonly used 40% threshold. This result suggested that no serious common method bias was indicated.

A total of 1,872 valid responses were included in the final analytic sample. The mean age was 20.31 years (SD = 1.43), and 59.4% of participants were female. The sample included students from humanities and social sciences, science and engineering, and medicine or health-related disciplines. GenAI use was common: 29.8% of students reported using GenAI 3–5 times per week, and 24.4% reported daily or almost daily use. The distribution of use frequency differed markedly across profiles, with daily or almost daily use concentrated in the high-risk dysregulated group.

Participant characteristics by latent profile are shown in Table 1. Gender and discipline did not differ significantly across profiles. Age and grade showed significant but modest differences, with the high-risk dysregulated profile including a slightly higher proportion of senior students than the low-risk adaptive profile.

**Table 1:** Participant characteristics by latent profile.

Variable	Category	Total *N* (%)	P1 Low-risk	P2 Anxiety-prone	P3 Sleep-disrupted	P4 High-risk	*p*
Profile size		1,872 (100.0)	746 (39.9)	482 (25.7)	371 (19.8)	273 (14.6)	
Gender	Male	760 (40.6)	302 (40.5)	190 (39.4)	146 (39.4)	122 (44.7)	0.412
	Female	1,112 (59.4)	444 (59.5)	292 (60.6)	225 (60.6)	151 (55.3)	
Age	Mean ± SD	20.31 ± 1.43	20.18 ± 1.36	20.34 ± 1.41	20.39 ± 1.46	20.55 ± 1.51	0.021
Grade	Freshman	485 (25.9)	220 (29.5)	123 (25.5)	84 (22.6)	58 (21.2)	< 0.001
	Sophomore	538 (28.7)	221 (29.6)	144 (29.9)	101 (27.2)	72 (26.4)	
	Junior	495 (26.4)	191 (25.6)	126 (26.1)	101 (27.2)	77 (28.2)	
	Senior or above	354 (18.9)	114 (15.3)	89 (18.5)	85 (22.9)	66 (24.2)	
Discipline	Humanities/social sciences	675 (36.1)	276 (37.0)	184 (38.2)	126 (34.0)	89 (32.6)	0.184
	Science/engineering	811 (43.3)	319 (42.8)	198 (41.1)	169 (45.6)	125 (45.8)	
	Medicine/health-related	386 (20.6)	151 (20.2)	100 (20.7)	76 (20.5)	59 (21.6)	
GenAI use frequency	Rarely	351 (18.8)	242 (32.4)	65 (13.5)	31 (8.4)	13 (4.8)	< 0.001
	1–2 times/week	507 (27.1)	257 (34.5)	159 (33.0)	64 (17.3)	27 (9.9)	
	3–5 times/week	558 (29.8)	182 (24.4)	173 (35.9)	134 (36.1)	69 (25.3)	
	Daily or almost daily	456 (24.4)	65 (8.7)	85 (17.6)	142 (38.3)	164 (60.1)	

Values are presented as *n* (%) unless otherwise indicated. Differences across latent profiles were examined using chi-square tests or one-way ANOVA.

GenAI use frequency differed clearly across profiles. Daily or almost daily use was reported by 8.7% of low-risk adaptive users and 60.1% of high-risk dysregulated users, but daily use also appeared in the two intermediate profiles.

Descriptive statistics, internal consistency coefficients, and bivariate correlations are presented in Table 2. Problematic GenAI use showed moderate positive correlations with academic anxiety (*r* = 0.43), sleep problems (*r* = 0.36), and psychological distress (*r* = 0.46), indicating that dysregulated GenAI use co-occurred with multiple mental health risk indicators. Health literacy was negatively correlated with problematic GenAI use (*r* = −0.31), academic anxiety (*r* = −0.27), sleep problems (*r* = −0.22), and psychological distress (*r* = −0.30). Self-regulated learning was also negatively related to risk indicators and positively related to academic engagement (*r* = 0.51). These correlations supported the subsequent person-centered and network analyses.

**Table 2:** Descriptive statistics, reliability, and correlations.

Variable	*M*	SD	Cronbach's α	1	2	3	4	5	6	7
1. Problematic GenAI use	2.62	0.80	0.91	–						
2. Academic anxiety	3.02	0.86	0.90	0.43^***^	–					
3. Sleep problems	6.17	2.83	0.81	0.36^***^	0.41^***^	–				
4. Psychological distress	2.56	0.76	0.88	0.46^***^	0.55^***^	0.44^***^	–			
5. Health literacy	3.13	0.58	0.89	−0.31^***^	−0.27^***^	−0.22^***^	−0.30^***^	–		
6. Self-regulated learning	3.30	0.64	0.92	−0.47^***^	−0.36^***^	−0.28^***^	−0.39^***^	0.42^***^	–	
7. Academic engagement	3.33	0.67	0.90	−0.28^***^	−0.25^***^	−0.20^***^	−0.34^***^	0.28^***^	0.51^***^	–

Sleep problems were measured using a PSQI-type score, with higher scores indicating poorer sleep quality. Other variables were measured on Likert-type scales. ^***^p < 0.001.

### 3.2 Identification of latent profiles

Fit indices for the one- to five-profile solutions are shown in Table 3. The one-profile model was retained only as a baseline model because it assumed a homogeneous student group. The two- and three-profile models improved the information criteria and yielded broad categories, but the three-profile model merged anxiety-prone and sleep-disrupted patterns into a general at-risk group. The four-profile model had lower AIC, BIC, and aBIC values than the three-profile model, high entropy (0.891), significant LMR and BLRT results, and adequate class sizes.

**Table 3:** Fit indices for latent profile models.

Model	AIC	BIC	aBIC	Entropy	LMR p	BLRT p	Smallest class %	Decision
1-profile	27,342.6	27,420.1	27,375.8	–	–	–	–	Baseline
2-profile	25,118.4	25,239.7	25,170.2	0.846	< 0.001	< 0.001	38.2	Candidate
3-profile	24,006.9	24,172.0	24,077.3	0.874	0.002	< 0.001	20.9	Candidate
4-profile	23,541.3	23,750.2	23,630.3	0.891	0.015	< 0.001	14.6	Preferred
5-profile	23,392.6	23,645.3	23,500.2	0.872	0.118	< 0.001	4.2	Not selected

AIC, Akaike information criterion; BIC, Bayesian information criterion; aBIC, sample-size adjusted BIC; LMR, Lo–Mendell–Rubin adjusted likelihood ratio test; BLRT, bootstrapped likelihood ratio test.

Although the five-profile model showed slightly lower information criteria, it generated a very small class representing only 4.2% of the sample, and the LMR test was not significant. This solution was therefore considered less stable and potentially overextracted. The four-profile solution was selected because it provided the best balance between statistical fit, classification precision, class size, and substantive interpretability. Average posterior classification probabilities ranged from 0.902 to 0.942, indicating good profile separation (Supplementary Table S2). Robustness checks for alternative profile solutions are presented in Supplementary Figure S3.

**Figure 2:**
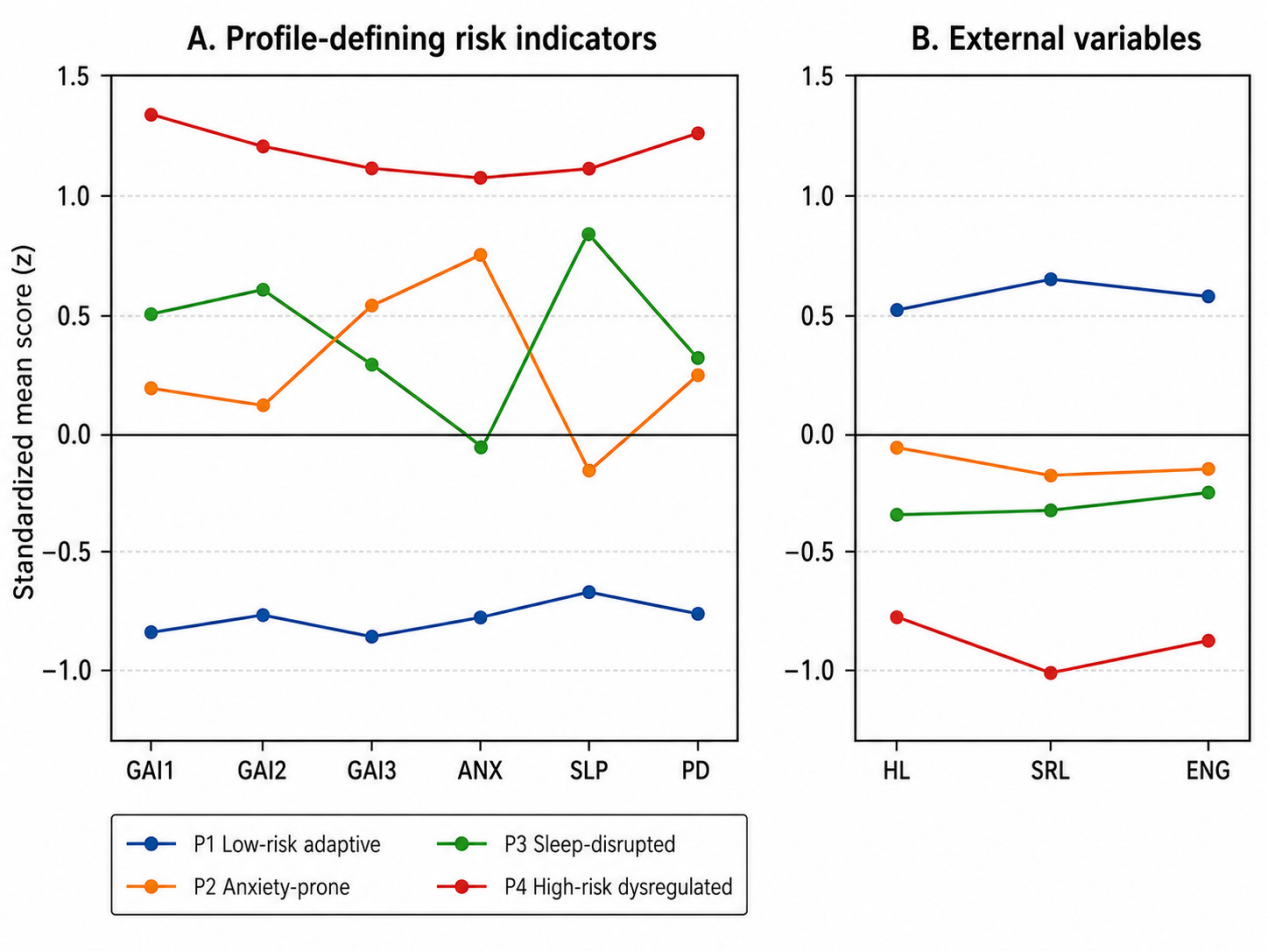
GenAI-use and mental-health risk profile patterns and external resource/functioning variables. **(A)** Standardized mean scores of the six indicators used in latent profile analysis. **(B)** Standardized mean scores of external resource and functioning variables across profiles. GAI1, loss of control; GAI2, compulsive GenAI use; GAI3, academic avoidance; ANX, academic anxiety; SLP, sleep problems; PD, psychological distress; HL, health literacy; SRL, self-regulated learning; ENG, academic engagement.

The four GenAI-use and mental-health risk profiles were labeled low-risk adaptive users, anxiety-prone dependent users, sleep-disrupted overusers, and high-risk dysregulated users. Standardized profile-defining indicators and external comparison variables are displayed separately in Figures 2A, B. Low-risk adaptive users (39.9%) showed low profile-defining indicators and higher external resource/functioning scores. Anxiety-prone dependent users (25.7%) showed elevated academic anxiety and academic avoidance. Sleep-disrupted overusers (19.8%) showed elevated GenAI-use dysregulation and sleep problems. High-risk dysregulated users (14.6%) showed high scores across profile-defining indicators and low scores across external resource and functioning variables.

### 3.3 Differences across latent profiles

Mean differences across profiles are reported in Table 4. Omnibus tests were significant for all core variables. Profile-defining indicators showed clear separation across groups: high-risk dysregulated users scored highest on loss of control, compulsive GenAI use, academic avoidance, academic anxiety, sleep problems, and psychological distress. They also had the lowest health literacy, self-regulated learning, and academic engagement.

**Table 4:** Differences in profile-defining indicators, modifiable resources, and academic functioning across latent profiles.

Variable	P1 Low-risk M ±SD	P2 Anxiety-prone M ±SD	P3 Sleep-disrupted M ±SD	P4 High-risk M ±SD	*F*	*p*	*Post hoc*
Problematic GenAI use	1.92 ± 0.43	2.79 ± 0.46	3.05 ± 0.49	3.67 ± 0.51	924.31	< 0.001	P4 > P3 > P2 > P1
Loss of control	1.81 ± 0.48	2.72 ± 0.52	3.02 ± 0.55	3.76 ± 0.57	876.44	< 0.001	P4 > P3 > P2 > P1
Compulsive GenAI use	2.03 ± 0.51	2.80 ± 0.53	3.20 ± 0.58	3.69 ± 0.60	693.18	< 0.001	P4 > P3 > P2 > P1
Academic avoidance	1.78 ± 0.49	3.05 ± 0.57	2.84 ± 0.61	3.55 ± 0.65	742.66	< 0.001	P4 > P2 > P3 > P1
Academic anxiety	2.25 ± 0.55	3.72 ± 0.61	2.94 ± 0.66	4.01 ± 0.58	851.29	< 0.001	P4 > P2 > P3 > P1
Sleep problems	4.11 ± 1.86	5.72 ± 2.03	8.56 ± 2.11	9.34 ± 2.28	604.75	< 0.001	P4 > P3 > P2 > P1
Psychological distress	1.96 ± 0.47	2.76 ± 0.56	2.81 ± 0.60	3.52 ± 0.64	581.40	< 0.001	P4 > P3/P2 > P1
Health literacy	3.42 ± 0.44	3.10 ± 0.48	2.91 ± 0.51	2.68 ± 0.55	212.63	< 0.001	P1 > P2 > P3 > P4
Self-regulated learning	3.75 ± 0.49	3.18 ± 0.55	3.04 ± 0.58	2.63 ± 0.61	349.82	< 0.001	P1 > P2 > P3 > P4
Academic engagement	3.72 ± 0.52	3.22 ± 0.59	3.14 ± 0.61	2.71 ± 0.64	268.47	< 0.001	P1 > P2/P3 > P4

Values are presented as mean ± standard deviation. *Post hoc* comparisons were conducted after omnibus tests. Health literacy, self-regulated learning, academic engagement, and problematic GenAI use total score were external comparison variables and were not used as profile-defining indicators. P1, low-risk adaptive users; P2, anxiety-prone dependent users; P3, sleep-disrupted overusers; P4, high-risk dysregulated users.

Low-risk adaptive users showed the opposite pattern, with the lowest profile-defining scores and the highest external resource and functioning scores. This profile indicates that GenAI exposure alone was not the defining signal.

The two intermediate profiles differed in configuration. Anxiety-prone dependent users had higher academic anxiety and academic avoidance than sleep-disrupted overusers, whereas sleep-disrupted overusers had higher sleep-problem scores. These differences support the separation of anxiety-driven and sleep-related risk patterns.

*Post hoc* comparisons further supported the profile labels. Problematic GenAI use total scores, reported as an external descriptive indicator, increased in the order P1 < P2 < P3 < P4. Health literacy and self-regulated learning decreased across the same sequence. Academic anxiety was higher in P2 than in P3, whereas sleep problems were higher in P3 than in P2.

### 3.4 Factors associated with profile membership

Table 5 presents the multinomial logistic regression results, with the high-risk dysregulated profile as the reference group. The model examined external factors associated with profile membership and did not include profile-defining indicators as predictors. Higher health literacy (OR = 2.41, 95% CI: 1.82–3.20), self-regulated learning (OR = 2.86, 95% CI: 2.11–3.87), and academic engagement (OR = 1.65, 95% CI: 1.25–2.18) were associated with greater odds of belonging to the low-risk adaptive profile rather than the high-risk dysregulated profile.

**Table 5:** Multinomial logistic regression examining factors associated with latent profile membership.

Predictor	P1 OR	95% CI	*p*	P2 OR	95% CI	*p*	P3 OR	95% CI	*p*
Female	1.12	0.84–1.49	0.441	1.09	0.80–1.49	0.580	1.03	0.75–1.42	0.842
Age	0.92	0.84–1.01	0.076	0.96	0.87–1.06	0.412	0.98	0.88–1.09	0.701
Daily GenAI use	0.38	0.31–0.46	< 0.001	0.53	0.43–0.65	< 0.001	0.71	0.58–0.87	0.001
Health literacy	2.41	1.82–3.20	< 0.001	1.54	1.15–2.07	0.004	1.38	1.03–1.86	0.032
Self-regulated learning	2.86	2.11–3.87	< 0.001	1.58	1.16–2.16	0.004	1.44	1.05–1.97	0.024
Academic engagement	1.65	1.25–2.18	< 0.001	1.31	0.98–1.75	0.069	1.22	0.91–1.63	0.181

Reference group, P4 High-risk dysregulated users; P1, low-risk adaptive users; P2, anxiety-prone dependent users; P3, sleep-disrupted overusers; OR, odds ratio; CI, confidence interval. Variables used to define latent profiles were not entered as predictors to avoid circular interpretation.

Daily or almost daily GenAI use was associated with lower odds of belonging to the low-risk adaptive profile (OR = 0.38, 95% CI: 0.31–0.46), anxiety-prone dependent profile (OR = 0.53, 95% CI: 0.43–0.65), or sleep-disrupted overuser profile (OR = 0.71, 95% CI: 0.58–0.87) rather than the high-risk dysregulated profile. Frequent use was therefore most concentrated in the high-risk group, but profile interpretation still depended on broader risk configuration.

Associations involving health literacy, self-regulated learning, and academic engagement were strongest for low-risk adaptive membership and smaller for the intermediate profiles. Academic engagement was clearly associated with low-risk membership but showed weaker associations with the intermediate profiles.

### 3.5 Network structure and bridge nodes

**Figure 3:**
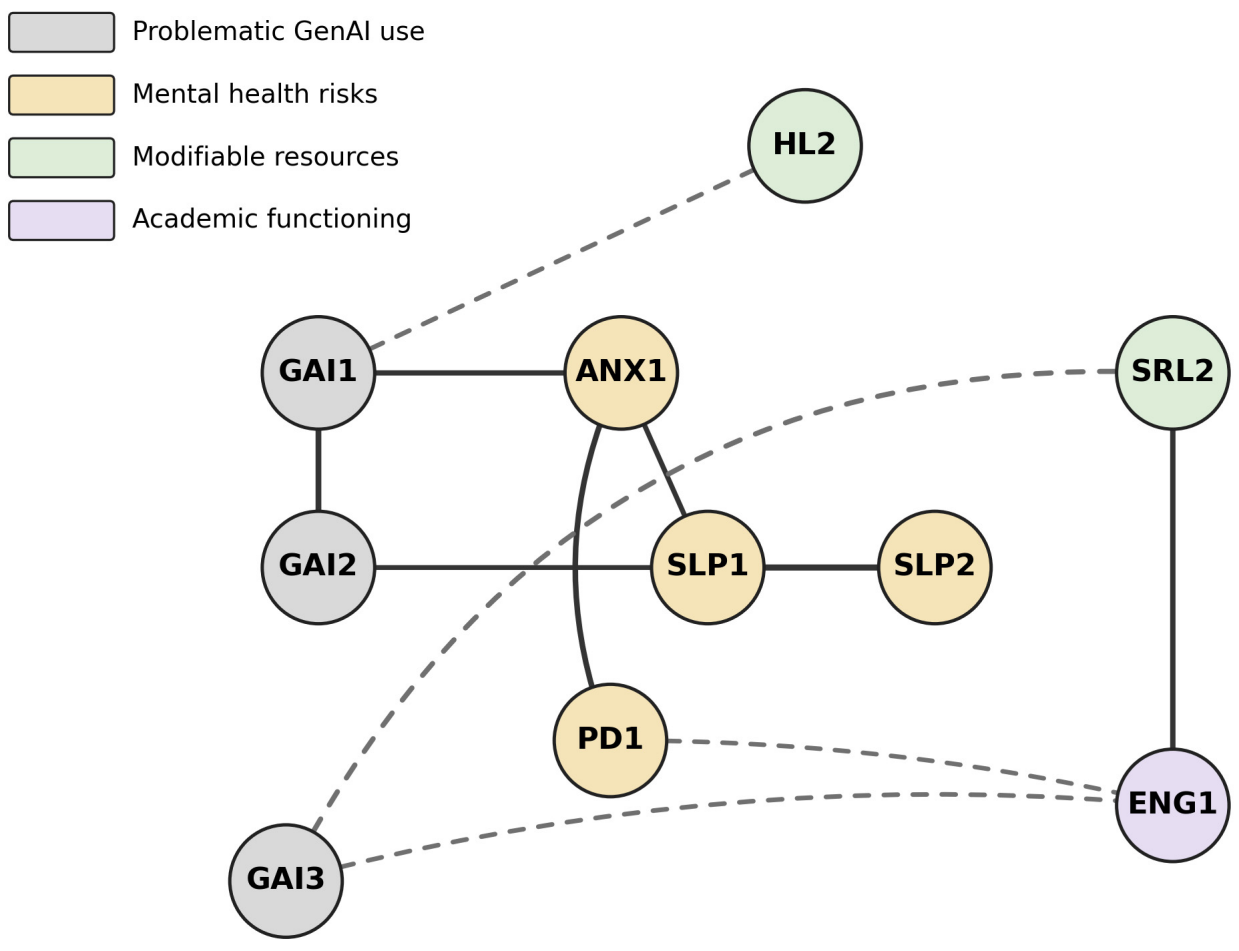
Network structure of problematic GenAI-use indicators, mental health indicators, modifiable resources, and academic functioning. The network was estimated using EBICglasso and visualized using qgraph. Solid edges indicate positive partial correlations, and dashed edges indicate negative partial correlations. GAI1, loss of control; GAI2, compulsive GenAI use; GAI3, academic avoidance; ANX1, academic worry; SLP1, delayed bedtime; SLP2, daytime dysfunction; PD1, emotional exhaustion; HL2, information credibility evaluation; SRL2, time management; ENG1, academic engagement.

The estimated EBICglasso network is shown in Figure 3. The strongest edges appeared within the sleep, problematic-use, and mental-health indicator clusters. The edge between delayed bedtime and daytime dysfunction was the strongest positive edge, followed by the edges between loss of control and compulsive GenAI use, and between academic worry and psychological distress. These associations indicate meaningful within-domain clustering rather than isolated indicators.

Several cross-domain edges were also notable. Loss of control was conditionally associated with academic worry, suggesting that difficulty regulating GenAI use co-occurred with academic concerns after other nodes were controlled. Compulsive GenAI use was conditionally associated with delayed bedtime, indicating a statistical link between repeated or late-night GenAI use and sleep-related indicators. Academic avoidance was negatively connected with self-regulated learning and academic engagement, suggesting that avoidance-oriented GenAI use may be linked to lower adaptive learning functioning.

**Figure 4:**
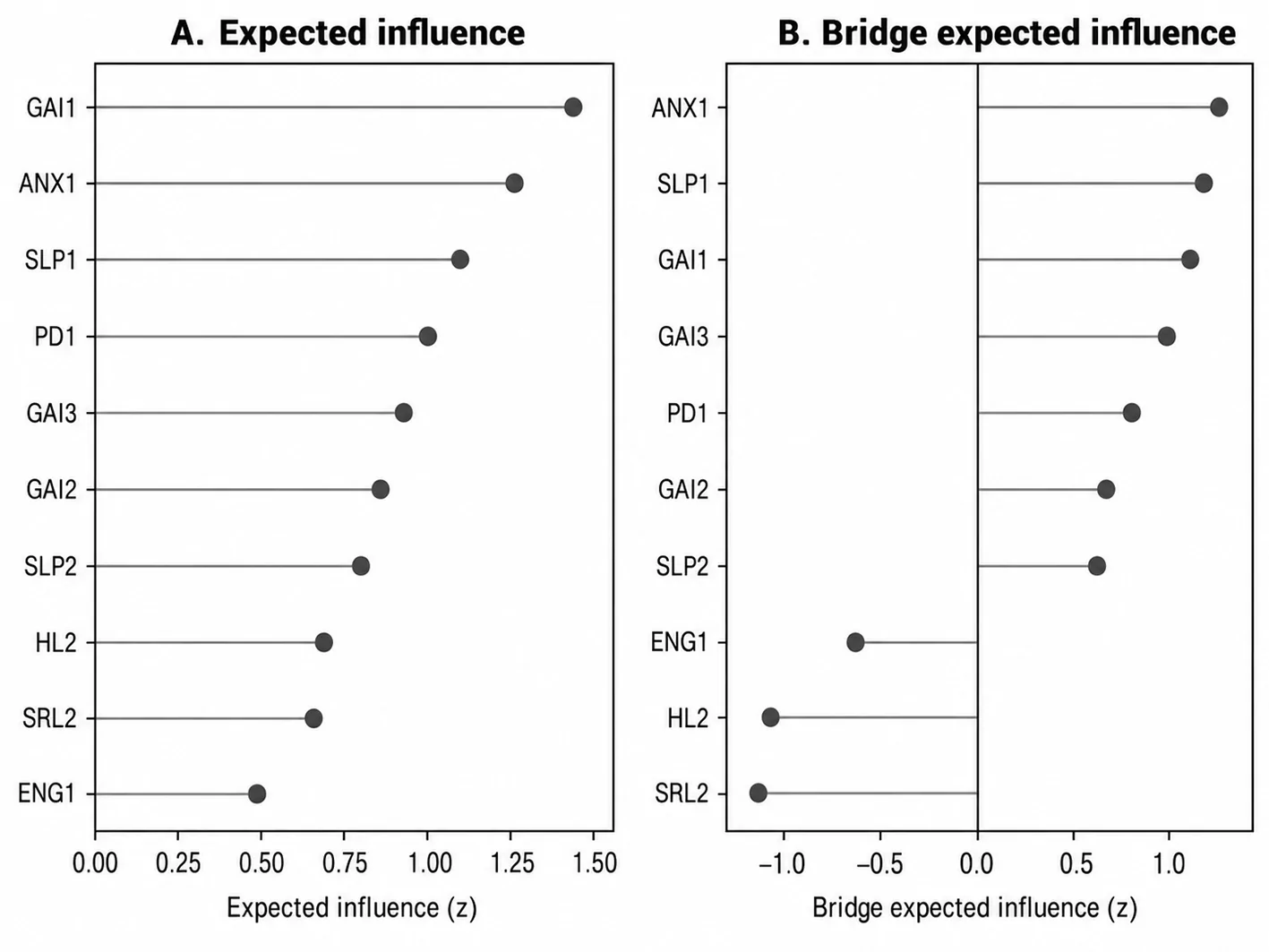
Expected influence and bridge expected influence of network nodes. **(A)** Expected influence indicates the overall centrality of each node in the network. **(B)** Bridge expected influence indicates the extent to which a node connects different conceptual communities. Positive values indicate stronger cross-community bridge positions, whereas negative values indicate inverse cross-community connections that were mainly observed for modifiable-resource nodes in this study. Values are standardized z-scores.

Figure 4 shows the expected influence and bridge expected influence indices. Loss of control, academic worry, delayed bedtime, and emotional exhaustion had the highest expected influence. Academic worry, delayed bedtime, loss of control, and academic avoidance had the highest positive bridge expected influence, suggesting that these nodes statistically connected problematic GenAI use with mental health indicators and academic functioning. In contrast, information credibility evaluation and time management showed negative bridge expected influence, indicating resource-related bridge positions in the network.

The supplementary network checks supported the stability of these findings. Bootstrapped edge-weight accuracy is shown in Supplementary Figure S1, and case-dropping bootstrap stability is shown in Supplementary Figure S2. Expected influence was highly stable, with a correlation stability coefficient of 0.750. Bridge expected influence also showed good stability, with a correlation stability coefficient of 0.596. These indices support interpretation of the network structure as an exploratory map of conditional associations.

## 4 Discussion

### 4.1 Principal findings

The main message is that frequent GenAI use alone was not the key public health signal. A more concerning pattern appeared when impaired control, academic worry, delayed bedtime, distress, and weaker self-regulatory resources co-occurred. The LPA separated four such configurations, and the regression results showed that health literacy and self-regulated learning were associated with lower-risk membership relative to the high-risk dysregulated profile. The network results then identified loss of control, academic worry, delayed bedtime, and academic avoidance as cross-domain nodes in this conditional association structure.

This pattern also avoids a simple good-or-bad account of GenAI use. Daily use was concentrated in the high-risk group, but intensive use did not by itself define the profile structure. Some students may use GenAI often for ordinary academic work; others appear to rely on it in contexts of worry, avoidance, and late-night study.

For campus practice, this means that blanket prohibition is unlikely to be the most informative response. Screening and support discussions may usefully attend to impaired control, coping motives, functional disruption, and modifiable skills. Because the data are cross-sectional, these implications should be read as prevention-oriented hypotheses rather than evidence that changing any single node would produce clinical improvement.

### 4.2 Interpretation of latent profiles

Low-risk adaptive users combined low risk with stronger health literacy, self-regulation, and engagement. This group should not be interpreted as avoiding GenAI. Rather, these students may use AI tools within clearer boundaries, verify information, and maintain independent learning routines. The implication is not to eliminate GenAI use, but to support regulated and informed use.

Anxiety-prone dependent users showed a different configuration. Their academic anxiety and academic avoidance were prominent, whereas sleep problems were less severe than in the sleep-disrupted group. For these students, GenAI may provide short-term reassurance, but the observed pattern is also consistent with avoidance of difficult learning activities and lower confidence in independent problem solving.

Sleep-disrupted overusers were marked by poor sleep and elevated GenAI-use dysregulation. This profile may reflect late-night AI-assisted studying, repeated revision of assignments, or difficulty disengaging from screens before bedtime. These interpretations remain associative and should be tested with longitudinal or diary-based designs.

High-risk dysregulated users showed the most severe configuration: high GenAI-use problems, academic anxiety, sleep problems, psychological distress, and low resource indicators. Cross-sectional data cannot establish temporal order, but the co-occurrence of these indicators points to a group that may warrant early monitoring and tailored campus support.

The adapted Problematic GenAI Use scale should therefore be interpreted as a research measure of GenAI-use dysregulation rather than a diagnostic instrument. Its items were designed to translate established problematic digital-use features into the GenAI context, including impaired control, compulsive checking, overreliance, academic avoidance, reduced independent thinking, emotional dependence, and excessive time use. The CFA, CR, AVE, and discriminant-validity results support its use in the present sample, but additional validation is needed before the construct can be treated as fully established across populations or platforms.

### 4.3 Network associations between GenAI dependence and mental-health indicators

The network results add specificity to the profile findings. Loss of control was a central problematic-use node, indicating that the capacity to stop, set boundaries, and preserve learning agency may be more informative than use frequency alone. This finding is associative and should not be interpreted as evidence that loss of control causes other problems.

Academic worry and delayed bedtime were important bridge nodes. Academic worry may statistically connect AI dependence with mental-health indicators through reassurance seeking, while delayed bedtime may connect digital behavior with sleep timing and daytime functioning. Because bridge expected influence is derived from a cross-sectional network, these nodes should be viewed as candidate indicators for future longitudinal or trial-based research rather than confirmed clinical targets.

The modifiable-resource nodes also deserve attention. Credibility evaluation and time management showed negative bridge expected influence, suggesting that information verification and planned AI use were inversely connected with the more difficult profile features. This pattern supports further testing of whether such skills consistently distinguish lower-risk GenAI-use and mental-health profiles in longitudinal data.

### 4.4 Public health implications

On this basis, responsible GenAI education could be folded into existing campus health-promotion work rather than treated only as an academic-integrity issue. The relevant content would include mental health literacy, sleep-hygiene reminders, information-evaluation skills, and help-seeking guidance. This risk-identification orientation is consistent with public health studies that use interpretable modeling, lifestyle-risk assessment, and problematic digital-use frameworks to identify candidate indicators for follow-up (51–53).

One cautious implication is that campus support could be considered according to the pattern of difficulties reported by students. Universal materials could cover responsible GenAI use, basic sleep-health guidance, and credibility checking. More focused support could be considered for students who report academic worry, late-night AI use, or difficulty reducing use. Students with high dysregulation and distress may require combined counseling, academic advising, and digital behavior planning, a model that should be tested prospectively before stronger recommendations are made. This approach aligns with campus-based health-promotion studies addressing emotion management, screen-media behavior, food-related behavior, physical activity, and other modifiable student health indicators (54–56). Such a cautious, staged approach is also consistent with meta-analytic evidence on universal mental health prevention and social-emotional learning programs for students (60, 61).

Digital health literacy programs also need to account for the way GenAI presents information. Students need to verify AI-generated content, recognize unsupported or hallucinated claims, and know when professional advice is needed. Because fluent AI responses can appear authoritative, students with lower health literacy may be more likely to report misplaced trust or overreliance. This interpretation is consistent with digital health and health-literacy frameworks emphasizing reliable information, informed decision-making, and reduced health inequalities in digital environments (57–59).

Person-centered screening should remain brief and exploratory at this stage. Items on loss of control, academic worry, delayed bedtime, academic avoidance, and difficulty reducing AI use could be tested in future longitudinal studies. Different patterns of difficulty may correspond to different forms of support, but the present findings do not establish intervention effects.

### 4.5 Strengths and limitations

This study links GenAI use with public health concerns among university students and combines person-centered and network approaches. LPA identified heterogeneous GenAI-use and mental-health risk profiles, whereas network analysis pointed to specific behaviors and symptoms that may be useful for future hypothesis testing. The sample size was adequate for the analytic approach, and the study included both profile-defining indicators and external modifiable-resource variables.

Several limitations should be noted. The cross-sectional design cannot establish causal relationships or temporal ordering; pre-existing anxiety, sleep problems, or distress may also precede dysregulated GenAI use. All measures were self-reported and may be affected by recall bias, social desirability, or common method variance. Although Harman's single-factor test did not indicate serious common method bias, this procedure cannot rule out all shared-method effects. The convenience sampling strategy and the focus on Chinese university students also limit generalizability. Future studies should combine self-report with behavioral logs, ecological momentary assessment, or objective sleep indicators when feasible.

The study did not examine institutional AI policies, teacher expectations, peer norms, or platform-specific differences. These contextual factors may be associated with whether GenAI use appears regulated or dysregulated. Future research could combine individual-level data with classroom or institutional variables and compare different GenAI platforms, tasks, and policy environments.

Finally, GenAI-related problematic use remains a developing measurement area. The adapted scale showed good reliability and acceptable CFA, CR, AVE, and discriminant-validity evidence in this sample, but this study was not a full scale-development project and did not test measurement invariance, test-retest reliability, predictive validity, or external behavioral criteria. Further validation across cultures, age groups, educational settings, and independent samples is needed. Network edges and centrality indices should also be interpreted as hypothesis-generating rather than causal proof. Generalizability beyond Chinese university students should be tested in future research.

## 5 Conclusion

Problematic GenAI use among university students is an emerging digital public health concern marked by heterogeneous GenAI-use and mental-health risk profiles. This study identified four profiles and showed that high-risk dysregulated users reported elevated GenAI-use problems, academic anxiety, sleep problems, and psychological distress, together with lower health literacy, self-regulated learning, and academic engagement. In the network, loss of control, academic worry, delayed bedtime, and academic avoidance were key nodes, while credibility evaluation and time management showed negative bridge expected influence. The findings point to a cautious public health response in which universities can monitor difficulty controlling AI use, anxiety-driven reliance, late-night AI use, and academic avoidance, while future studies test whether strengthening digital health literacy, self-regulation, and sleep-health practices is associated with changes in GenAI-use patterns and student well-being.

## Data Availability

The raw data supporting the conclusions of this article will be made available by the authors, without undue reservation.
