# Supplementary material for: Problematic generative AI use and mental health risks among Chinese university students: latent profiles, network structure, and health literacy as a modifiable resource
Source: Front Public Health. 2026 Aug 20;14:1910408. doi: 10.3389/fpubh.2026.1910408 (PMC13538084; doi:10.3389/fpubh.2026.1910408)
Supplement: Supplementary file 1 [file Supplementary_file_1.docx]

**Supplementary Material**

**Table S1. Measurement information and scale sources.**

| **Construct** | **Items / indicators** | **Measurement focus** | **Response format** | **Score interpretation** | **Source / adaptation** | **Cronbach’s α** |
| --- | --- | --- | --- | --- | --- | --- |
| Problematic GenAI use | 8 items; LPA indicators: loss of control, compulsive use, academic avoidance | Loss of control, failed reduction, compulsive checking, overreliance, academic avoidance, reduced independent thinking, emotional dependence, excessive time use | 5-point Likert scale | Higher scores indicate more dysregulated GenAI use | Adapted from problematic Internet use, generalized problematic Internet use, smartphone overuse, and technology-use frameworks; wording revised for GenAI context | 0.91 |
| Academic anxiety | 5 items | Academic worry, fear of falling behind, assignment/exam anxiety, nervousness during difficult academic tasks | 5-point Likert scale | Higher scores indicate greater academic anxiety | Adapted from student academic-anxiety and mental-health research | 0.90 |
| Sleep problems | 7 PSQI-type domains | Subjective sleep quality, sleep latency, sleep duration, sleep efficiency, sleep disturbance, daytime dysfunction, delayed bedtime related to digital-device or GenAI use | PSQI-type scoring | Higher scores indicate poorer sleep quality and more sleep-related problems | Adapted from PSQI-type sleep-health and digital-use studies | 0.81 |
| Psychological distress | 6 items | Emotional exhaustion, mental overload, difficulty relaxing, irritability, reduced motivation, perceived inability to cope | 5-point Likert scale | Higher scores indicate greater psychological distress | Selected to capture broader distress indicators common in university populations | 0.88 |
| Health literacy | 8 items | Accessing, understanding, evaluating, and applying health information; judging reliability of online/AI-mediated information | 4-point response scale | Higher scores indicate stronger health literacy | Based on health-literacy definitions and short-form health literacy approaches | 0.89 |
| Self-regulated learning | 6 items | Goal setting, time management, monitoring progress, strategy adjustment, independent problem solving before seeking AI help | 5-point Likert scale | Higher scores indicate stronger self-regulated learning | Adapted from self-regulated learning frameworks | 0.92 |
| Academic engagement | 6 items | Behavioral, cognitive, emotional, and interactive engagement | 5-point Likert scale | Higher scores indicate greater academic engagement | Adapted from academic engagement research | 0.90 |

Note. GenAI = generative artificial intelligence; PSQI = Pittsburgh Sleep Quality Index; LPA = latent profile analysis.

**Table S2. Average posterior classification probabilities for the four-profile solution.**

| **Most likely profile** | **n** | **%** | **P1 Low-risk adaptive users** | **P2 Anxiety-prone dependent users** | **P3 Sleep-disrupted overusers** | **P4 High-risk dysregulated users** |
| --- | --- | --- | --- | --- | --- | --- |
| P1 Low-risk adaptive users | 746 | 39.9 | 0.942 | 0.032 | 0.015 | 0.011 |
| P2 Anxiety-prone dependent users | 482 | 25.7 | 0.025 | 0.918 | 0.034 | 0.023 |
| P3 Sleep-disrupted overusers | 371 | 19.8 | 0.018 | 0.044 | 0.902 | 0.036 |
| P4 High-risk dysregulated users | 273 | 14.6 | 0.010 | 0.023 | 0.025 | 0.942 |

Note. Values on the diagonal represent average posterior probabilities for the assigned profile.

**Table S3. Psychometric evidence for adapted and selected scales.**

| **Construct** | **Items** | **Standardized loading range** | **Cronbach’s α** | **CR** | **AVE** |
| --- | --- | --- | --- | --- | --- |
| Problematic GenAI use | 8 | 0.68–0.84 | 0.91 | 0.92 | 0.58 |
| Academic anxiety | 5 | 0.72–0.86 | 0.90 | 0.90 | 0.64 |
| Psychological distress | 6 | 0.66–0.81 | 0.88 | 0.89 | 0.57 |
| Health literacy | 8 | 0.63–0.80 | 0.89 | 0.90 | 0.53 |
| Self-regulated learning | 6 | 0.74–0.88 | 0.92 | 0.92 | 0.66 |
| Academic engagement | 6 | 0.70–0.85 | 0.90 | 0.91 | 0.62 |

Note. The confirmatory factor analysis showed acceptable model fit: χ²/df = 3.42, CFI = 0.958, TLI = 0.951, RMSEA = 0.036, and SRMR = 0.041. CR = composite reliability; AVE = average variance extracted. The square roots of AVE were higher than the corresponding inter-construct correlations, supporting discriminant validity.

**Table S4. Data-quality screening and excluded responses.**

| **Step** | **Screening criterion** | **Excluded n** | **Remaining n** | **Operational details** |
| --- | --- | --- | --- | --- |
| Initial submitted questionnaires | Survey submitted after consent | -- | 2,140 | Questionnaires submitted through the online survey platform |
| 1 | Incomplete questionnaire | 96 | 2,044 | Questionnaires missing non-core information or showing incomplete submission records |
| 2 | Failed attention-check item | 54 | 1,990 | One embedded attention-check item; responses failing this item were excluded |
| 3 | Completion time < 180 seconds | 68 | 1,922 | Threshold based on pilot completion time and platform response-time logs |
| 4 | Patterned responses | 50 | 1,872 | Identical answers across long item blocks or visually repetitive response strings screened using platform export, SPSS, and R checks |
| Final analytic sample | Valid responses retained for analysis | -- | 1,872 | Effective response rate = 87.5% |

**Figure S1. Accuracy of network edge weights based on nonparametric bootstrapping. Points indicate estimated edge weights, and horizontal lines indicate bootstrapped 95% confidence intervals.**

**Figure S2. Case-dropping bootstrap stability of expected influence and bridge expected influence. The expected influence index remained highly stable, and bridge expected influence also showed good stability.**

**Figure S3. Robustness checks for alternative latent profile solutions. The four-profile solution provided the best balance of statistical fit, entropy, class size, and substantive interpretability.**
